# Deoxygenation lowers the thermal threshold of coral bleaching

**DOI:** 10.1038/s41598-022-22604-3

**Published:** 2022-10-31

**Authors:** Rachel Alderdice, Gabriela Perna, Anny Cárdenas, Benjamin C. C. Hume, Martin Wolf, Michael Kühl, Mathieu Pernice, David J. Suggett, Christian R. Voolstra

**Affiliations:** 1 Climate Change Cluster, Faculty of Science, University of Technology Sydney, Ultimo, NSW 2007 Australia; 2 Department of Biology, University of Konstanz, 78457 Konstanz, Germany; 3 Marine Biology Section, Department of Biology, University of Copenhagen, Strandpromenaden 5, 3000 Helsingør, Denmark

**Keywords:** Genetics, Molecular biology

## Abstract

Exposure to deoxygenation from climate warming and pollution is emerging as a contributing factor of coral bleaching and mortality. However, the combined effects of heating and deoxygenation on bleaching susceptibility remain unknown. Here, we employed short-term thermal stress assays to show that deoxygenated seawater can lower the thermal limit of an *Acropora* coral by as much as 1 °C or 0.4 °C based on bleaching index scores or dark-acclimated photosynthetic efficiencies, respectively. Using RNA-Seq, we show similar stress responses to heat with and without deoxygenated seawater, both activating putative key genes of the hypoxia-inducible factor response system indicative of cellular hypoxia. We also detect distinct deoxygenation responses, including a disruption of O_2_-dependent photo-reception/-protection, redox status, and activation of an immune response prior to the onset of bleaching. Thus, corals are even more vulnerable when faced with heat stress in deoxygenated waters. This highlights the need to integrate dissolved O_2_ measurements into global monitoring programs of coral reefs.

## Introduction

Oceans are deoxygenating under climate warming, to the extent that the global ocean dissolved O_2_ (DO) content is predicted to decline by as much as 7% by 2100^1,2^. Drastic consequences are already apparent where key marine ecosystems are experiencing an increased frequency of insufficient O_2_ for normal physiological functioning, i.e. hypoxia, that drive swift widespread mortality^3^ developing new—or expanding existing—dead zones^4–6^. Indeed, exposure to deoxygenation and resultant hypoxia is rapidly emerging as a key contributing agent of mass coral bleaching-induced mortality worldwide^6–8^, with episodes now documented on Caribbean reefs in Panama^6,9,10^ and in the Gulf of Mexico^11^. Here, DO of tropical reef waters have reduced to ≤ 2 mg L^−1^ from the combined effects of coastal nutrient loading that amplify biological O_2_ demand and seasonal heating that lowers O_2_ water solubility, driving severe deoxygenation events. Given that coral reefs are already present in more than half of the known dead zones, there are likely many more undocumented critical deoxygenation events on reefs^6^, and hypoxia must be more extensively considered as a threat to coral survival^7,8^. Intriguingly, reef-building coral populations have been documented to thrive under extremely low O_2_ conditions in deeper water^12,13^, in hot and acidic mangrove environments^14,15^, and through summers in one of the warmest seas, the Persian Arabian Gulf^16^. Such studies highlight the capacity of corals in particular habitats to tolerate hypoxia alongside other environmental conditions that would normally drive corals to bleach^17–19^. However, in contrast to decades of effort to unlock the interactive role of temperature and pH on coral bleaching^20–22^, synergistic effects of temperature and deoxygenation remain almost entirely unexplored.

Whilst reef-building corals have recently been shown to exhibit the capacity to oxy-regulate under both hypoxic^23^ and near anoxic^24^ conditions, coral bleaching and mortality can be induced when exposed to deoxygenation^25–27^. Similarly to other stressors such as heat or acidification^28–31^, hypoxia-induced bleaching susceptibility appears highly variable between coral species, most likely due to acclimation and adaptation differences to reduced O_2_ availability (e.g.,^25,32^). For example, susceptibility of shallow water tropical corals to deoxygenation stress has been observed for *Acropora yongei* when exposed to 2–4 mg L^−1^ O_2_ for 72 h^27^, *Acropora cervicornis* under 0.5 mg L^−1^ O_2_ for 24 h^26^, and *Acropora selago* with 2 mg L^−1^ O_2_ for 12 h during the night^25^. In contrast, relatively high hypoxia tolerance thresholds have been observed for *Orbicella faveolata* after ~ 10 days under severely deoxygenated conditions (~ 0.5 mg L^−1^ O_2_;^26^). Interestingly, *Acropora tenuis,* ascribed as more heat stress tolerant than *Acropora selago*^33^, also exhibited a greater tolerance to deoxygenation with no signs of bleaching when exposed to ~ 2 mg L^−1^ O_2_ for 12 h^25^. This latter study revealed that corals possess a complete Hypoxia-Inducible Factor-mediated Hypoxia Response System (HIF-HRS)—a key gene network for hypoxia stress mitigation in metazoans—and found the bleaching of *Acropora selago* to align with a lower capacity to upregulate HIF target genes. Such target genes are involved in key processes including shifting to anaerobic respiration or gluconeogenesis, a reduction in mitochondrial activity, or an increase in protein quality control, lipid resourcing, cell apoptosis, and antioxidant activity^25,32,34^. Interestingly, these processes have also been reported in coral under heat stress^17,29,35–42^ suggesting transcriptomic commonalities under these stressors^22^ and indicating that deoxygenation would amplify the stress responses we see under heat, but it remains to be explicitly tested.

As with high light and high temperature stress for corals^22,43,44^, hypoxia is also known to induce oxidative stress in metazoans by modifying mitochondrial activity to result in decreased ATP synthesis and increased ROS (reactive oxygen species) production^45^. Our recent studies have demonstrated that gene expression profiles of corals exposed to deoxygenation show evidence for reduced mitochondrial complex I activity and enhanced formation of ROS-handling molecules^12,25,34^. Importantly, enhanced ROS and RNS (reactive nitrogen species) production has been indicated to be an important signal, along with low O_2_, to activate the HIF-HRS^46^. Thus, it is entirely plausible that hypoxia may be an underlying trigger of oxidative cellular stress in bleaching coral.

Here we investigated how deoxygenation combined with heat stress impacts the coral bleaching thermal threshold of a key reef-building *Acropora* species using the Coral Bleaching Automated Stress System (CBASS;^47^. This system has recently been used to resolve differences in coral thermotolerance via short-term acute heat stress assays that correspond to physiological differences under long-term heat stress^48,49^ or geographically distant sites in the Red Sea^29,50^. We applied CBASS for the first time under both normoxic (6 mg L^−1^ O_2_) and lowered oxygen concentrations (2 mg L^−1^ O_2_) to (i) mimic heat stress under deoxygenation, and (ii) demonstrate how CBASS assays can be employed to assess the effects of different stressors in concert and in isolation. Both bleaching index scores and measurements of photosynthetic efficiency, i.e., maximum PSII quantum yield, were then used to quantify whether deoxygenation can lower the coral thermal threshold by means of ED50 scores, i.e. standardized thermal thresholds^49^. We performed RNA-Seq analysis on treated coral fragments to demonstrate how deoxygenation stress can induce mitochondrial dysfunction, enhanced oxidative stress, and a reduced ability to sense, signal, and protect against photooxidative damage—contributing to overall coral bleaching susceptibility.

## Materials and methods

### Coral samples

All coral fragments were derived from the same parent colony (a single genet) of a ‘Blue Staghorn’ undetermined species of *Acropora* (hereafter, *Acropora* sp.), which had been propagated in a mesocosm aquarium tank (at B&B Aquakultur, Konstanz, Germany) under consistent parameters for > 10 years (Table S1) to conceive a zero ecological footprint coral experiment. Fragments can therefore be considered genetically identical and appropriate for experimental examination of putative interactive heat and deoxygenation stress effects for this colony and hence bleaching phenotype (sensu^25^). The undetermined species was originally sourced off Bali, Indonesia in 2010, where the maximum monthly mean (MMM) sea surface temperature (SST) is ~ 30 °C. Experimental coral fragments were fragmented from the parent colony in two subsequent batches (a first CBASS run without deoxygenation H0 and then a second run with deoxygenation Hd) a week prior to CBASS assaying. A total of 32 fragments ranging from 2 to 4 cm in length were used for each experimental CBASS run and positioned upright on ceramic plugs (Figure S1). Coral nubbins were positioned in a rack in seawater in a polystyrene box for the short transportation from aquaria rearing facilities to the University of Konstanz for CBASS assaying (31st May 2021, heat only, H0; 4th June 2021, heat with deoxygenation, Hd).

### Experimental setup

We used the Coral Bleaching Automated Stress System (CBASS) to conduct short-term acute heat stress assays (as described in Voolstra et al.^29,47^), modified to incorporate a seawater reservoir to finely regulate dissolved O_2_ (DO) and pH that enters the CBASS (see below). The system was set up in a wet laboratory (University of Konstanz) near the aquarium holding tanks to minimise coral transport time. Briefly, the core CBASS consisted of four replicate 10L flow‐through tanks equipped each with a temperature controller, chillers, a heater, and a 165 W full spectrum LED aquarium light (Figure S1). Temperature profiles for each tank were programmed by a temperature controller (InkBird ITC-310T-B) connected to 2 thermoelectric chillers (IceProbe T, Nova Tec) and 1 aquarium heater (Titanium, 200W; Schego). For each tank, photon irradiance was delivered by a dimmable 165 W full spectrum LED aquarium light (Galaxyhydro) to match the rearing aquaria light fields (~ 240 μmol photons m^−2^ s^−1^ using half blue and half white LED spectral range intensities), as quantified using an Apogee MQ-510 Underwater Quantum meter. In each tank, a HOBO pendant temperature logger was positioned on the opposite side to the heater to provide consistent temperature recordings throughout the experiment (Figure S2). Regulation of O_2_ and pH levels was through a 140L sealed seawater reservoir connected to CO_2_, compressed air, and O_2_ gas cylinders. Customised gas regulators were fitted to adjust the DO and CO_2_ entering the seawater through aquarium air stones. DO and pH probes (WTW Multi 3630 IDS) were introduced into a pipe of the flowing reservoir water to constantly read O_2_ and pH to finely regulate gas contributions. The reservoir was first flushed with N_2_ to lower DO to < 2 mg L^−1^, where after CO_2_ was flushed through the deoxygenated seawater to offset changes in pH, as described in Alderdice et al. (2021). DO and pH in the reservoir and experimental tanks were also measured hourly during the 6 h heat stress phase (Figure S3) using robust O_2_ and pH probes (OXROB10 and PHROB-PK8, Pyroscience) connected to a FireStingPro fibre-optic multi-meter (Pyroscience). We note that the starting levels of pH between each CBASS run (H0 and Hd) were largely identical (± < 0.05). Similarly, inherent lowering of pH levels by 0.2 units under heating is within the natural diel range experienced by corals on reefs^30,51^ as well as within the diel range in the holding aquarium (Table S1). Water in the reservoir was pre-heated to the control/baseline temperature with aquarium heaters (Titanium, 200W; Schego) linked to a programmable temperature controller (InkBird ITC-310T-B). Before the start of the experiment, the four CBASS tanks were slowly filled with pre-heated deoxygenated seawater to avoid re-oxygenation until starting of the actual CBASS assays. A transparent lid was placed over experimental tanks to reduce the air space above the water surface, and any potential air gaps were sealed using Sanitop-Wingenroth ‘plastic-fermit’ (Figure S1). Seawater from the reservoir was delivered during the CBASS assays by the flow-through system at a flow rate of 25 mL min^−1^ to achieve seawater turnover (1.5 L/hour) for each experimental tank. Seawater for long-term aquaria rearing and in the CBASS assays had a salinity of 35 PSU and was prepared using the same salt (Fauna Marin, Germany).

**Figure 1:**
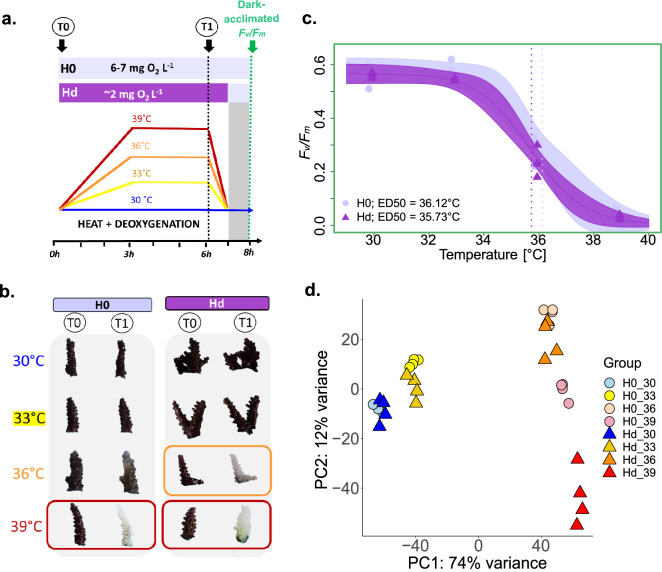
Deoxygenation lowers the thermal threshold of coral bleaching. (**a)** Short-term thermal stress assay profiles with respective 3 h heat-hold temperatures at 30 °C (control/baseline), 33 °C, 36 °C, and 39 °C under normoxic (H0) and deoxygenation (Hd) conditions. For Hd, the deoxygenation condition was applied for a total of 6 h (during daylight hours). Samples for RNA-Seq analysis were taken after 6 h, i.e. at the end of the heat-hold as indicated by T1. Measurements of maximum photosynthetic efficiency of PSII (*F*_*v*_*/F*_*m*_) of the additional fragments were taken following 1 h dark-acclimation. **(b)** Representative photographs of coral fragments from time point T0 and T1 under H0 and Hd. **(c)** Maximum photosynthetic efficiency of PSII (*F*_*v*_*/F*_*m*_) in relation to temperature for H0 vs. Hd (n = 3 for each) and determined ED50 thermal tolerance thresholds as a proxy for coral bleaching susceptibility (sensu^29,49^). Solid lines reflect the log-logistic model with 95% confidence intervals represented by the shaded areas. **(d)** Principal Component Analysis (PCA) of 20,115 transcripts comparing all H0 and Hd samples across all heat stress temperatures (n = 4 for each). The x- and y-axes indicate the percent of the variance explained by the first and second principal component, respectively.

Out of the 4 CBASS tanks, a control tank was maintained at 30 °C for the duration of the experiment to exhibit the maximum monthly mean (MMM) temperature of the parent colony origin (Bali, Indonesia; MMM approximately 28.5–29.5 °C pending on exact sampling location) which was determined from the NOAA Coral Reef Watch 5 km database^52^. MMM was used as the baseline/control temperature rather than the long-term aquarium rearing temperature of 27 °C. This is in line with the notion that corals exhibit locally adapted thermal thresholds^50,53,54^ and following recently established CBASS protocols in which short term assays were shown to be representative of longer-term experiments^48,49^. In support of this notion, we trialled an initial CBASS run where the baseline temperature was set to a lower temperature of 27 °C and observed a projected ED50 temperature threshold (ED50 = 36.05 °C) that exceeded the highest treatment temperature (36 °C); conventionally, the ED50 should ideally lie below the highest treatment temperature for prediction accuracy (Figure S4). Our trial emphasizes how corals seem to retain their evolutionarily acquired thermal threshold, even under long-term aquaria rearing conditions; this further corroborates previous CBASS studies that use MMM as the baseline/control temperature^47,49,50^. Using the MMM temperature (30 °C) as the baseline/control, we then applied heat treatments to the other 3 tanks accordingly (sensu^47,49^) at 33 °C (MMM +3 °C), 36 °C (MMM +6 °C), and 39 °C (MMM +9 °C). All temperature treatments were heated over a 3 h period, held for another 3 h at target temperature, and then decreased back to baseline (30 °C) over 1 h, where they were retained for the remainder of the 18 h experiment (Fig. 1a; Figure S2). For the combined heat and deoxygenation CBASS run, we used deoxygenated seawater of ~ 2 mg L^−1^ O_2_ during the heating ramp and heat-hold but allowed for re-oxygenation during the overnight recovery phase (Figure S3). Fragments from all CBASS assays were sampled for RNA-Seq by flash-freezing in liquid N_2_ after the 6 h heat stress phase to capture the transcriptional response to either only heating (H0) or to heating and deoxygenation (Hd). Dark-acclimated photosynthetic efficiencies, i.e. the maximum PSII quantum yield (*F*_*v*_/*F*_*m*_), were measured on the remaining fragments following 1 h dark-acclimation at the start of the overnight recovery phase. As such, for both CBASS runs (H0 and Hd), a total of 7 clonal ramets were assayed in each temperature tank, 4 used for RNA-Seq analysis (sampled after 6 h of the CBASS profiles) and the remaining 3 used for measuring dark-acclimated *F*_*v*_/*F*_*m*_ (measured after 7 h, Fig. 1).

### Coral bleaching assessment

Measurements of the dark‐acclimated maximum photosynthetic efficiency of photosystem II (*F*_*v*_/*F*_*m*_) were used to determine ED50-based thermal tolerance limits as a proxy for coral bleaching (sensu^29,49^). Photosynthetic efficiency was previously found to be most indicative of coral  thermal tolerance when comparing coral whitening, chlorophyll a, host protein, algal symbiont counts, and algal type association^47^. For all *F*_*v*_/*F*_*m*_ measurements, coral fragments were retained under darkness for at least 1 h prior to measurements using a pulse-amplitude modulated fluorometer (Mini PAM II; Walz). One measurement per fragment was taken to avoid PAM saturating light pulses potentially inducing light artefacts. Measurements were consistently taken on the mid side of the upright fragment to avoid the tip or base of the fragment that would have experienced different light exposure (e.g.,^55^), putatively influencing *F*_*v*_/*F*_*m*_ values. Optimized PAM settings (following^56^) were used as follows: Signal Gain and Damping of 2, Measuring Light Curve Intensity (Mi) of 5, Actinic Light Factor of 1, Saturation Intensity and Width of 3 and 0.8, respectively. Of note, recorded readings were taken when the minimum fluorescence (F_0_) was > 200 (instrument units) on fragments. Temperature tolerance thresholds were determined for both H0 and Hd as the mean temperature (across all ramets) at which *F*_*v*_/*F*_*m*_ dropped to 50% of the value measured at baseline temperatures, here defined as the Effective Dose 50 or ED50^49^ using the DRC package in R^57^. Statistical differences among treatment-specific ED50s were assessed via a Welch’s unequal variances (one-tailed) t-test with replicate-based treatment ED50s as the response variable and treatment as the respective factor. The script and data are available at https://github.com/reefgenomics/CBASS_hypoxia. Since all fragments were sourced from a single parent colony, we are testing for a significant treatment effect between ramets of a genet. Hence, the true effect size might be larger if response is measured across biological population replicates.

Coral fragments were photographed using an Olympus TG-6 digital camera alongside a ‘Coral Watch Coral Health Chart’ colour reference for visual bleaching assessments, before being flash-frozen in liquid N_2_ and stored at – 80 °C until further processing. Photographs were taken after the 6 h stress phase (T1) and also prior to the CBASS experiment at T0 using the same camera settings, working distance, and illumination level (all images are available at https://zenodo.org/record/6497221;^58^). We devised a bleaching index score (BIS) based on the previously established ‘colour score’^59^ and ‘relative bleaching score’^60^. For this, colour saturation of the tissue for each coral fragment was ranked on a 6-point scale via visual assessment to the colorimetric reference card with 6 representing maximum saturation and 1 representing no pigment (i.e., completely white), respectively. Bleaching was considered to have occurred when a decrease in colour saturation of two or more values was observed prior or after the stress exposure^59^. Instances where we found inconsistent coloration across the fragment, two scores (one for the paler part and one for the more colourful part) were determined and averaged. Bleaching Index Scores (BIS) were determined by 5 independent assessors, who did not have prior knowledge of the experiment and who scored the coral fragments in a randomised manner. We chose to include BIS, as BIS accounts for a coral holobiont compound phenotype (i.e. colour), whereas *F*_*v*_*/F*_*m*_ relates to the photochemical efficiency of the algal symbionts and may not encompass host-specific effects to deoxygenation. Standardized thermal temperature thresholds were determined as for *F*_*v*_/*F*_*m*_, but this time ED50 scores designated the inferred temperature at which the BIS dropped to or below 50% of the value at baseline temperatures (ED50;^29,49^) using the DRC package in R^57^. Statistical differences among treatment-specific ED50s were assessed via a Welch’s unequal variances t-test (one-tailed) with individual treatment ED50s as the response variable and treatment as the factor.

### RNA extraction and sequencing

Total RNA was extracted using the Qiagen RNeasy mini kit using the QIAcube Connect. Frozen coral fragments were immersed in RLT buffer (Qiagen) within zip-lock bags and tissue was air-picked from the skeleton using airflow from a sterile, 1,000 µL pipette tip connected via a rubber hose to a benchtop air pressure valve for a maximum of 3 min. A 400 µL aliquot of the resulting tissue slurry was centrifuged at full speed for 3 min and then 350 µL of the supernatant of each respective sample were loaded into 2 mL tubes and inserted into the QIAcube Connect to run the RNeasy extraction protocol. RNA concentrations were assessed using the Qubit RNA Broad-Range assay kit on the Qubit 4 fluorometer (Invitrogen). RNA quality was evaluated by capillary electrophoresis via the presence of intact 18S and 28S ribosomal RNA bands using the QIAxcel RNA quality control kit v2.0 on the QIAxcel Advanced system (Qiagen). Total RNA was shipped to the sequencing facility on dry ice. RNA from each sample was used to generate 2 × 150 bp paired-end libraries. Sequencing was performed on the NovaSeq 6000 sequencer at Novogene, UK. RNA-Seq data are available under NCBI BioProject PRJNA808230 (available at https://www.ncbi.nlm.nih.gov/bioproject/PRJNA808230).

### De novo transcriptome assembly

Demultiplexed reads were quality-checked using FASTQC^61^ before and after read trimming with trimmomatic v0.38^62^ to remove Illumina adapters, low quality reads, and reads shorter than 50 bp. Each sample retained > 96% of the paired-end read counts (Table S2). For an initial analysis, a de novo transcriptome was assembled using SOAPdenovo-Trans using the default 23-kmer length^63^ with reads from all samples. To gauge the proportion of assembled contigs across taxa of the coral holobiont (e.g., Cnidaria, Dinophyceae, bacteria, virus, and fungi), the transcriptome was queried against the BLASTn database. Given the relatively high proportion of contigs assigned to bacteria (Table S3), the transcriptome was re-assembled using only reads of non-bleached samples (i.e. excluding 36 °C and 39 °C samples), which lowered the proportion of bacteria contigs from 16 to 5%. The transcriptome was further filtered to only consider contigs with a length ≥ 500 bp, comprising a total of 20,115 contigs of which 17,975 could be assigned to Cnidaria^58^. To assess the number of distinct cnidarian loci, we clustered the 17,975 contigs assigned to Cnidaria with a similarity threshold of 90% using CD-HIT-EST^64^. This procedure returned 17,960 transcripts, suggesting that the majority of cnidarian contigs reflects distinct genes. The assembly comprising only non-bleached samples also had a higher scaffold N50 of 1,946 compared to 1,442 for all samples (considering contigs ≥ 500 bp (Table S4). We consider the transcriptome of 20,115 contigs the reference transcriptome on which all expression analyses were based on, accessible at https://zenodo.org/record/6497221^58^; we did not remove putative non-cnidarian loci to account for cross-mappings of RNA-Seq reads. For gene expression analyses, trimmed paired-end reads from all samples were mapped to the reference transcriptome using Bowtie 2 v2.3.5.1^65^. Resultant mapping files were processed with SAMtools^66^ to generate bam files to quantify mapped contigs (i.e., transcripts) using Salmon^67^. All samples had at least 5 million mapped reads for read 1 and so were included in the downstream analysis (Table S2).

### RNA-Seq analysis

Tximport^68^ was used to import count data from Salmon into the R environment for transcript-level count estimation. To visualise general patterns of gene expression, variance-stabilising transformed counts were used for the Principal Component Analysis (PCA), plotted using the R package ggplot2^69^. Count data was then filtered to include only cnidarian transcripts for differential expression analysis using the package DESeq2^70^. The number of reads assigned to Dinophyceae was insufficient to perform differential expression analysis. Normalization for sequencing depth was applied through the DESeq2 dispersion function. Wald testing for significance difference of coefficients with a negative binomial general linear model (GLM) was applied in DESeq2. *P* values were corrected using Benjamini–Hochberg (BH) at a default false discovery rate (FDR) cut-off of 0.05 (Table S5, Table S6). Venn diagrams of the common and unique differentially expressed (DE) transcripts between comparisons were created using the R package ggVennDiagram^71^. The EggNOG v5.0 ortholog database by EMBL was used to annotate the reference transcriptome^72^. To assess DE transcripts annotated to genes commonly involved in the coral heat stress response, DE transcripts were screened for annotations associated with the following processes: calcium homeostasis, heat shock proteins (protein homeostasis), cytoskeleton rearrangement, cell death, mitochondria activity suppression (or glycolysis promotion), and increased reactive O_2_ and N_2_ species (summarised by^17^. Shared DE transcripts across conditions (determined by Venn diagrams) were screened against the list of heat stress associated genes of interest (see above) and the log2 fold change was visualized across comparisons in a heat map in the R package ggplot2. Genes also reported from coral hypoxia stress responses were indicated using the symbol “*” in the heat map^25,34^. FPKMs (i.e., fragments per kilobase per million mapped reads) were estimated using DESeq2 for genes known to stabilise or suppress HIFα protein. These genes are key to activating the hypoxia stress-mitigating gene network and were plotted using the R package ggplot2 to assess expression patterns across temperatures under normoxic (H0) or deoxygenated (Hd) conditions. FPKM expression estimates were summed up for transcripts that shared the same annotation (e.g., HSP90) sensu ^25,34^. Gene ontology (GO) enrichment analyses for all DE transcript lists were performed using the R package TopGO^73^ with a recommended weighted-Fisher *P* value cut-off of < 0.001. All scripts can be accessed on GitHub at https://github.com/reefgenomics/CBASS_hypoxia.

### DNA extractions and Symbiodiniaceae ITS2 amplification

Total DNA was extracted from all baseline/control samples (n = 8) using the DNeasy blood & tissue kit with the QIAcube Connect. For each sample, a 180 μL aliquot of the remaining coral tissue slurry in RLT buffer (see above) was incubated at 56 °C for 1 h with 20 μL proteinase K. Samples were then loaded into the QIAcube Connect to run the DNeasy blood & tissue protocol. DNA concentrations were assessed using a NanoDrop 2000c spectrophotometer. Amplification of the Symbiodiniaceae ITS2 region for each sample was achieved using the Qiagen Multiplex PCR kit, with 10–50 ng of DNA, and the primers SYM_VAR_5.8S2 [5′-GAATTGCAGAACTCCGTGAACC-3′] and SYM_VAR_REV [5′-CGGGTTCWCTTGTYTGACTTCATGC-3′]^74–76^ with unique 8-mer barcodes at the respective 5′ ends of each primer at a final primer concentration of 0.5 μM in a reaction volume of 10 μL. Thermal cycler conditions for ITS2 PCR amplifications consisted of an initial denaturation at 95 °C for 15 min, 35 cycles of 95 °C for 30 s, 56 °C for 90 s, and 72 °C for 30 s, followed by a final extension step of 72 °C at 10 min. To confirm successful amplification, 1 µL of each PCR product were run on a 1% agarose gel. Samples were cleaned using ExoProStar 1‐step (GE Healthcare) and normalized using the SequalPrep Normalization Plate Kit (ThermoFisherScientific). Barcoded samples were then pooled into a single 1.5 mL Eppendorf tube (17 μL per sample) and concentrated using a SpeedVac (Concentrator plus, Eppendorf). Quantification was done using Qubit (Qubit dsDNA High Sensitivity Assay Kit, Invitrogen). Samples were sequenced at 2 × 250 bp on the NovaSeq 6000 platform at the Novogene Sequencing Centre (Cambridge, England).

### ITS2-based Symbiodinaceae profiling

ITS2 sequences were submitted to SymPortal for quality control and ITS2 type profile analysis (https://symportal.org) as described in^77^. In brief, Symbiodiniaceae genera^78^ were identified through BLAST querying a database containing representatives of each Symbiodiniaceae genus and subgeneric ITS2 type profiles were designated by SymPortal based on the presence and abundance of the ITS2 sequences across samples and within the SymPortal database. These profiles were characterized by unique combinations of defining intragenomic variants (DIVs). Output data from SymPortal was then plotted in R. SymPortal output files are available at https://zenodo.org/record/6497221^58^.

## Results

### Deoxygenation lowers the thermal threshold of coral bleaching

To assess the effect of deoxygenation on coral bleaching, we ran short-term heat stress assays using the CBASS under normoxic (6 mg L^−1^ O_2_) and deoxygenated (2 mg L^−1^ O_2_) conditions. We subsequently determined the effective dose 50 (ED50) thermal threshold to obtain a standardised measure of coral thermal tolerance based on either BIS (termed ED50_BIS_) or measured photosynthetic efficiencies (termed ED50_*Fv/Fm*_). Based on BIS, coral fragments exhibited a greater extent of paling at lower temperatures when exposed to combined heating & deoxygenation conditions (Hd) compared to heating alone (H0), whereby ED50_BIS_ was 1.09 °C lower under Hd  compared to H0 alone (ED50_BIS_ Hd = 36.02 °C ± 0.39 vs. H0 = 37.11 °C ± 0.1 [mean ± SE], respectively; Figure S5). This difference was significant despite the small sample size and temporal constraints of short-term assays to manifest treatment differences (t = 2.41, df = 4.9, *P* value = 0.03). Similarly, ED50_*Fv/Fm*_ thresholds were lower for Hd (35.73 °C ± 0.15, [mean ± SE]) compared to H0 (36.12 °C ± 0.18), but only by 0.4 °C (t = 1.12, df = 3.98, *P *value = 0.16). Notably, ED50 thermal thresholds for this *Acropora* species were ~ 6–7 °C above the maximum monthly mean (MMM) temperature of the site of origin (Bali, Indonesia: ~ 30 °C). It is important to highlight that these ED50s are not an indicator of in situ thermal thresholds, but rather a diagnostic proxy of thermal limits based on 18-h thermal profiles. ITS2-based Symbiodiniaceae profiling using the SymPortal analytical framework^77^ confirmed uniform symbiont algal assemblage across samples, with samples associated with the two genera—*Cladocopium* (C21), which comprised the majority of sequences, and *Symbiodinium* (A1; Figure S6).

To explore for any consistencies or differences between normoxic and deoxygenated heat stress, we evaluated the expression profiles of 20,115 assembled transcripts using RNA-Seq. Principal component analysis revealed that samples were largely separated by the heat stress temperature, with PC1 explaining 74% of the variation (Fig. 1d). In addition, we found sub-clustering of samples at 30 °C and 33 °C versus 36 °C and 39 °C (Fig. 1 c, d). Further, samples at 39 °C under deoxygenated heat stress conditions were most distinct and separated from 39 °C heat stress samples suggesting that deoxygenation exerts a more pronounced effect under high heat stress. Lastly, greater dispersion of samples, i.e. a higher variance, was consistently demonstrated by heat and deoxygenation samples compared to heat only samples across all temperatures.

### Corals exhibit a similar response to heat stress with and without deoxygenation

**Figure 2:**
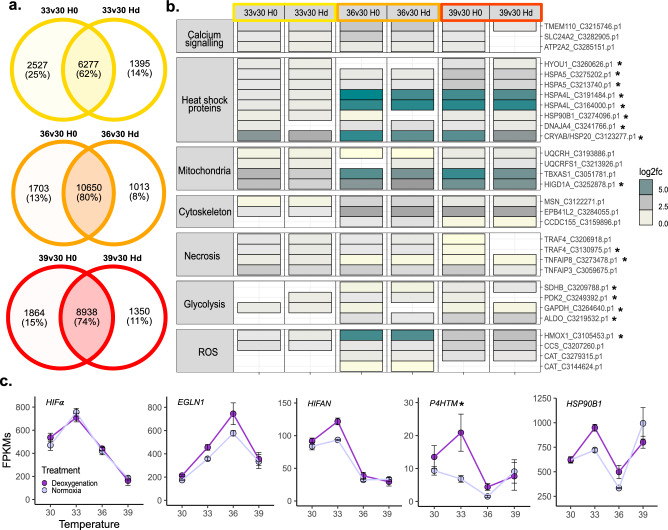
Corals exhibit a similar response to heat stress with and without deoxygenation. (**a)** Number (and percentage) of differentially expressed (DE) transcripts at FDR < 0.05 that were common to or unique between normoxic (H0) and deoxygenated (Hd) conditions when comparing heating temperatures (33 °C, 36 °C, 39 °C) to the baseline (30 °C). **(b)** Log_2_-fold change (FC_log2_) differences in gene expression of common heat stress genes under normoxic and deoxygenated heat stress. Considered DE transcripts are associated with calcium signalling, heat shock proteins, mitochondria, cytoskeletal restructuring, necrosis, glycolysis promotion, and ROS mitigation. Colour gradient indicates log_2_-fold change, where green and yellow represent a positive and negative fold change, respectively. White space indicates no DE. Asterisks indicate those transcripts also reported under deoxygenation stress in coral^25,34^. **(c)** Expression dynamics across temperatures for both conditions (H0 and Hd) for hypoxia-inducible factor alpha subunit (*HIFα*), prolyl hydroxylase domain 2/4 (*EGLN1*/*P4HTM*), HIFα inhibitor (*HIFAN*), and heat shock protein 90 (*HSP90B1*). Expression estimates are based on fragments per kilobase of transcript per million mapped reads (FPKM). Error bars denote standard error with n = 4 for each condition.

To elucidate how the heat stress response of corals exposed to heating (H0) compares to heating & deoxygenation (Hd), we assessed differential expression between each heating temperature (33 °C, 36 °C, 39 °C) and the baseline temperature of 30 °C for the normoxic and deoxygenated CBASS runs. Most differentially expressed (DE) transcripts (FDR < 0.05) were common between H0 and Hd at all heating temperatures with 62%, 80%, and 74% being shared at 33 °C, 36 °C, and 39 °C, respectively (Fig. 2a). Both the common and the total number of DE transcripts highlighted similar magnitudes of expression response for H0 and Hd, reflecting the PCA clustering predominantly by temperature irrespective of oxygen levels (Fig. 1d).

Among the commonly expressed EggNOG-annotated transcripts, many were involved in processes typically associated with coral heat stress responses including calcium homeostasis, heat shock proteins (protein homeostasis), cytoskeleton rearrangement, cell death, mitochondrial activity suppression, and increased RNS and ROS^17^. Of these, we found similar log_2_-fold changes for H0 and Hd at each heating temperature; for example, heat shock protein 70 (*HSPA4L*) exhibited greater log_2_-fold changes with higher temperatures (Fig. 2). We note that approximately half of the examined genes typically expressed under heat stress are shown to be activated under deoxygenation stress alone for other species of *Acropora*^25,34^. This includes the HIF-target genes pyruvate dehydrogenase kinase (*PDK2*) that promotes anaerobic respiration via the glucose to lactate pathway as well as heme oxygenase (*HMOX1*) that assists antioxidant activities (indicated by asterisks in Fig. 2b). Further, fragments per kilobase per million mapped reads (FPKM)-based expression estimates showed that the O_2_-sensitive hypoxia-inducible factor subunit (*HIFα*) and genes which either suppress (prolyl hydroxylase domain 2/4, *EGLN1*/*P4HTM*; HIFα inhibitor, *HIFAN*) or stabilise (heat shock protein 90; *HSP90B1*) *HIFα* proteins all followed similar expression dynamics under H0 and Hd conditions, except for *P4HTM*, which was significantly higher in Hd than H0 at 33 °C (log_2_-fold change (FC_log2_) = 1.66, FDR < 0.05). Although not significantly differentially expressed, the other two suppressors, *EGLN1* and *HIFAN*, exhibited greater expression under Hd at 33 °C and 36 °C. Notably, compared to the baseline temperature, *EGLN1* expression was significantly higher for both 33 °C and 36 °C under normoxic or deoxygenated conditions (33 °C vs 30 °C for H0 FC_log2_ = 1.10, for Hd FC_log2_ = 1.03; 36 °C vs 30 °C for H0 FC_log2_ = 1.83, for Hd FC_log2_ = 1.71, FDR < 0.05).

### Deoxygenation-specific stress responses may influence coral bleaching susceptibility

**Figure 3:**
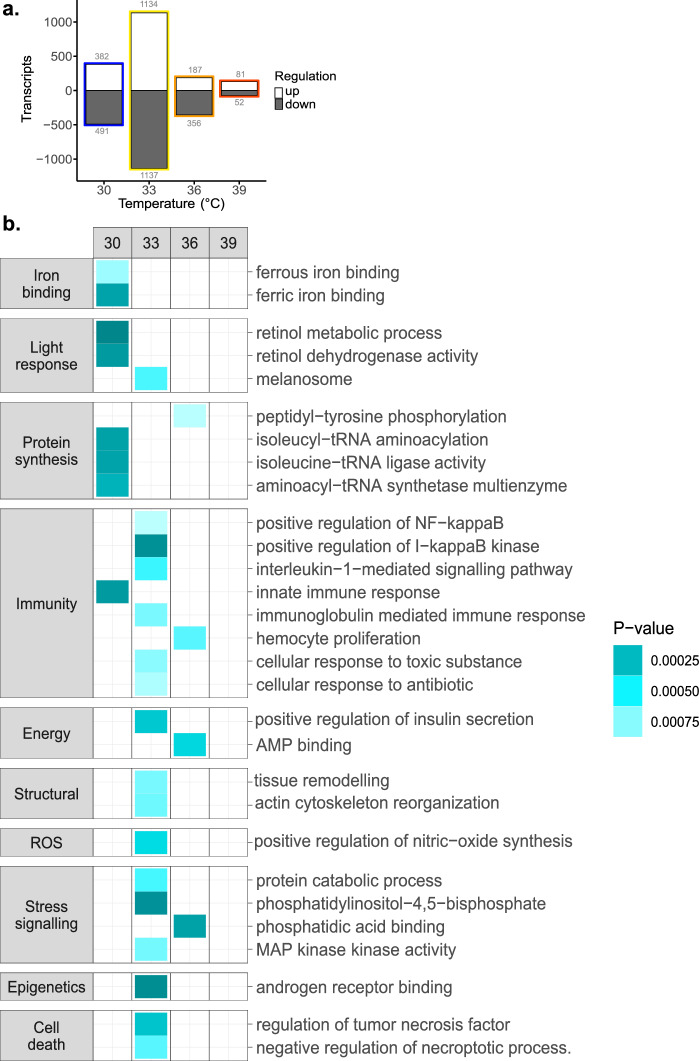
Deoxygenation-specific stress responses may influence coral bleaching susceptibility. (**a)** Number of differentially expressed transcripts that were up- (white) and down- (grey) regulated. At each temperature (30 °C, 33 °C, 36 °C, and 39 °C) deoxygenation and normoxic conditions are compared after 6 h of the thermal profile (time point 1, Fig. 1a). **(b)** Heat map of gene ontology (GO) enriched terms of differentially expressed transcripts (P-value < 0.001). Comparisons (top panel) include heat & hypoxia (Hd) versus heat only (H0) within temperatures (30 °C, 33 °C, 36 °C, and 39 °C). Categories (left) manually curated for the GO terms (right). The colour gradient indicates *P* values, where darker turquoise is indicative of greater significance. White space indicates no GO enrichment.

To understand how deoxygenation affects the coral heat stress response, we directly compared gene expression under normoxia (H0) and deoxygenation (Hd) at each temperature. Interestingly, the difference was greatest at 33 °C—with a total of 2271 DE transcripts – and became reduced with increasing temperature (Fig. 3). PCA plots of temperature-specific comparisons exhibited clear clustering of normoxia (H0) and deoxygenation (Hd) samples, with the Hd samples exhibiting a larger variance on average (Figure S7). Examination of DE transcripts via gene ontology (GO) enrichment analysis revealed categorial expression patterns across temperatures (Fig. 3). By means of the DE transcripts, samples at baseline temperature (30 °C) demonstrated a change in photosensitivity and an immune response, whereas samples under + 3 °C were mostly characterised by immune and stress signalling responses under deoxygenation, with little GO enrichment at higher temperatures (Fig. 3).

Deoxygenation samples expressed transcripts annotated in association with a response to light (retinol dehydrogenase activity; GO:0004745), iron binding (ferric iron binding; GO:0008199), protein synthesis (isoleucine-tRNA ligase activity; GO:0004822), and immunity (innate immune response; GO:0002758). Examples of gene regulation included retinol dehydrogenase (RDH5 FC_log2_ = -1.19, FDR < 0.05), which is involved in photoreceptive reactions, and the iron-binding procollagen-lysine 2-oxoglutarate 5-dioxygenase 1 (PLOD1 FC_log2_ = -1.05, FDR < 0.05) were both downregulated, whereas isoleucyl-tRNA aminoacylation (IARS FC_log2_ = 0.58, FDR < 0.05) and nuclear factor NF-kappaB (NFKB1 FC_log2_ = 0.37, FDR < 0.05) involved in protein synthesis and innate immunity signalling, respectively, were significantly upregulated. Of note, there were also significantly upregulated transcripts annotated to proteins associated with ROS-handling such as sulfite oxidase, peroxisomal biogenesis factor 11 beta, peroxidase, d-amino acid oxidase, and glutathione peroxidase (FC_log2_ = 0.74, 0.68, 0.5, 3.66, and 0.58 respectively, FDR < 0.05). Under + 3 °C heating (33 °C), deoxygenated samples were mostly enriched with GO terms associated with immune responses (e.g., immunoglobulin mediated; GO:0016064) or stress signalling (e.g., MAP kinase activity; GO:0004708), while others included cell death (regulation of tumour necrosis factor; GO:0010803), cytoskeletal restructuring (actin cytoskeleton reorganization; GO:0031532), energy regulation (positive regulation of insulin secretion; GO:0032024), ROS activity (positive regulation of NO synthase; GO:0051770), and a response to light (melanosome; GO:0042470).

We note that the phototransduction pigment rhodopsin was significantly downregulated at both 30 °C and 33 °C under heat & hypoxia (FC_log2_ = -0.63 and -1.42, FDR < 0.05, respectively). Further, at 30 °C the associated photoreceptive retinal pigment epithelial-specific 65 kDa protein was also downregulated (RPE65 FC_log2_ = -0.79, FDR < 0.05). In contrast, the photoprotective pigment, green fluorescent protein, was upregulated in transcript expression at 33 °C (GFP FC_log2_ = 0.82, FDR < 0.05), but downregulated compared to the baseline temperature irrespective of oxygen conditions (GFP Hd FC_log2_ = −0.93 vs H0 FC_log2_ = −1.73, FDR < 0.05). Furthermore, another photo-protective pigment, melanin, with the associated GO term melanosome was enriched under which genes involved in transport were upregulated at 33 °C (e.g., MYO5V FC_log2_ = 0.30, FDR < 0.05). In corroboration with the reduced number of DE transcripts at 36 °C there were few GO enriched terms except for those associated with an immune response (haemocyte proliferation; GO:0035172), stress signalling (phosphatidic acid binding; GO:0070300), and energy regulation (AMP binding; GO:0016208). At 39 °C, there was no GO enrichment of DE transcripts found. Taken together, these results highlight deoxygenation-specific responses to photo-reception, photo-protection, redox imbalance, and immunity.

## Discussion

Coral bleaching is a highly networked biological process, which reflects the outcome of multi-level and -scale stress exposures^22,38^. However, how the coral heat stress response is affected by O_2_ availability is not well understood, despite reefs becoming increasingly subject to deoxygenation under ocean warming—including water column stratification—and elevated biological O_2_ demands^1,2,8^. The Coral Bleaching Automated Stress System (CBASS) is a standardised system^47^ that allows for explicit testing of how individual and/or combined environmental factors affect thermal stress tolerance (i.e., bleaching) by assessing coral functioning under heat stress alone and in combination with an additional stressor, in this case deoxygenation. In line with recent CBASS studies ^29,47–49,79^, we extended the application of this approach to detect subtle, yet important differences in thermal sensitivity under differing O_2_ levels. Specifically, we found deoxygenation to lower the thermal bleaching threshold in *Acropora* coral by 1 °C and 0.4 °C according to bleaching index score- or photosynthetic efficiency-based ED50 thermal threshold modelling, respectively. Such difference is remarkable in the context of the constraints of a short-acute thermal stress assay based on 18-h thermal profiles and suggest that physiological impacts from altered O_2_ availability can manifest rather rapidly under heat stress. Whilst hypoxia stress-induced genes key to the HIF-HRS are differentially expressed under heat stress alone, a distinct deoxygenation response is also evident.

### Deoxygenation lowers thermal thresholds of coral bleaching

Previous studies have demonstrated that deoxygenation alone can drive coral bleaching and subsequent mortality^25,26^, suggesting that accelerating ocean deoxygenation under climate change and eutrophication could drastically impact the ability of corals – as aerobic metazoans – to respond to heat stress^7,22,24,25^. Based on established coral bleaching proxies of BIS^59,60^ and *F*_*v*_/*F*_*m*_ (e.g.,^80^, the Effective Dose 50 (ED50;^47,49^) metric affirms that deoxygenation lowers thermal thresholds for the species of *Acropora* examined here. Under 36 °C heating stress and deoxygenation, coral samples (Hd) exhibited greater visual paling with a lower BIS and a reduction in the ED50_BIS_ and ED50_*Fv/Fm*_ by 1 °C and 0.4 °C, respectively, compared to normoxic conditions (Fig. 1, Figure S5). With current climate models predicting at least a + 1.5 °C warming^81,82^, the lowering of the coral thermal threshold by a whole 1 °C (ED50_BIS_) under deoxygenation is a drastic result that warrants careful consideration in projecting the future survival of coral reefs.

It is important to note that the ED50_*Fv/Fm*_ (in contrast to the ED50_BIS_) was not significantly different between O_2_ treatments, which might be partially attributed to the low replicate number and the inherent constraints of short-term assays, where any measured response variable has to exhibit differences within 6 h. However, significance levels were much lower for ED50_*Fv/Fm*_ compared to ED50_BIS_, likely also reflecting that BIS accounts for a coral holobiont compound phenotype (i.e. colour), whereas *F*_*v*_*/F*_*m*_ relates to the photochemical efficiency of the algal symbiont and may not encompass host-specific effects to deoxygenation. Whilst temperature was the main driver of the transcriptional response, we found a subtle yet equally important difference in response to environmental O_2_ levels that needs to be accounted for when researching factors determining coral bleaching susceptibility (Fig. 1d). However, more experiments will be needed to ensure that our observations on the impact of deoxygenation on coral stress thermal thresholds are robustly drawn out. In particular, whether the observed relative minor difference in ED50 may be related to a relatively “mild”, i.e. sublethal rather than lethal, deoxygenation stress of 2 mg L^−1^ O_2_. Here, dose response experiments of both O_2_ and heat need to consider recent evidence for variable ‘oxy-regulation’ capacity amongst coral species that inherently determines the poise between sublethal and lethal physiological responses to reduced O_2_ availability^23^.

Under heating, the projected ED50_*Fv/Fm*_ thermal threshold values for our *Acropora* sp. were about 6 °C higher than the MMM temperature of the source of origin (ED50_H0_ = 36.12 °C, MMM ~ 30 °C). This is similar to previous observations for *Acropora cervicornis* (Florida; ED50_*Fv/Fm*_ = 35.88 °C, MMM = 30 °C;^79^), but lower than for *Stylophora pistillata* from the Central Red Sea (ED50_*Fv/Fm*_ = 38.27 °C, MMM = 30.75 °C;^29^), considered to be among the most thermally resilient corals^83^. Our results therefore add to the growing evidence that CBASS can resolve differences in thermal susceptibility across different taxa^29,47,50^ or environments^29,47,49,^ Notably, caution needs to be exercised that the ED50 thermal tolerance thresholds do not equate to long-term in situ thermal resilience, but rather to thermal limits within the framework of 18-h short term acute heat stress assays.

### Corals exhibit a similar response to heat stress with and without deoxygenation

In agreement with the overall expression patterns (Fig. 1d), a large percentage (62–80%) of differentially expressed genes was consistently observed under normoxic and deoxygenated heat stress (Fig. 2). Such similar magnitudes of expression difference likely highlight how the response to heating is associated with the response to hypoxia stress^35–37^. Notably, a common gene repertoire of stress response genes was previously proposed, where corals subjected to deoxygenation alone expressed genes typical of heat stress, such as different heat shock proteins and those involved in altering mitochondrial activity, lipid uptake, immunity, structural reorganisation, ROS-handling proteins, and cell apoptosis^17,25,34^. In line with this, we found the key hypoxia-induced transcriptional factor, *HIFα*, and its suppressor, *EGLN1/PHD2*, to be expressed under normoxic heat stress at lower temperatures (30 °C and 33 °C), which followed a similar expression pattern under deoxygenation heat stress. Consistent with our previous studies of deoxygenation stress alone^25,34^, *Acropora* sp. exhibited an increased expression of *EGLN1 (PHD2)* with increasing temperature, supporting the notion that overexpression of *EGLN1/PHD2* could signal for greater susceptibility to hypoxia or heat stress-induced bleaching. By comparison, an increased *PHD* expression under prolonged hypoxia stress is known to reactivate proteasomal degradation of *HIFα* by using intracellular O_2_ that is no longer being consumed in mitochondrial aerobic respiration^84^. Thus, the ability of *HIFα* and *PHD2* to register cellular hypoxia is desensitised, acting as a negative feedback mechanism of the HIF-HRS to protect cells against excessive cell death in an attempt to adjust to the hypoxic state^84,85^. As such, previous heat stress studies have likely also inherently reported a conflated response to deoxygenation, where corals shifted into a highly O_2_-demanding stress response exceeding the O_2_ supply. Tolerance to hypoxia stress in corals may thus ultimately both contribute and correspond to tolerance to heat stress, an outcome recently suggested where corals commonly ascribed to have greater heat stress tolerance (e.g., *Porites lutea*) possess a greater gene copy number of hypoxia stress-associated genes^32^. Coral samples from our analysis were associated with a Symbiodiniacae commonly found in species of *Acropora* (*Cladocopium*-dominated; C21), although the relevance of this is currently unknown. A key aspect still missing is the contribution of other coral holobiont members to deoxygenation. For instance, associated microbes may be consuming large amounts of O_2_ or shifting to a community of anaerobes, both of which could alter the overall O_2_ budget of the coral holobiont^86–88^. Further, high microbial O_2_ consumption associated with excessive algae production on coral reefs can lead to deoxygenation of the reef and consequential changes in the microbial and benthic communities^10,89^.

### Deoxygenation induces coral vulnerability to light- and heat-stress

**Figure 4:**
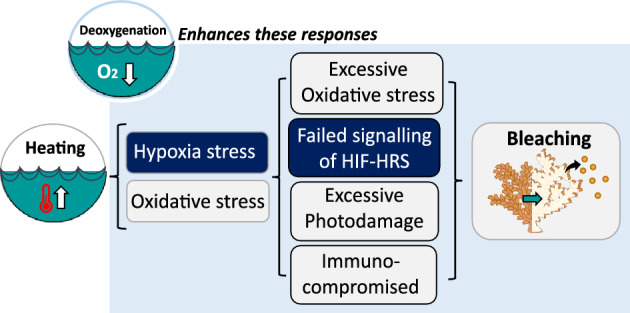
Incorporating hypoxia stress into the coral bleaching response. ROS accumulation is commonly observed in heat stressed corals, although it is unclear whether it is the actual trigger or a ‘smoking gun’, i.e. downstream of further upstream events that initiate the symbiotic breakdown^22,97^. Despite such uncertainties, hypoxia stress during heating may play a pivotal role in determining coral bleaching susceptibility. Specifically, hypoxia- and heat-driven excessive oxidative stress could hamper signalling of the Hypoxia-inducible Factor (HIF) hypoxia response system (HRS), enhance photodamage, and leave the coral immune-compromised. Increasing deoxygenation may thus increase coral bleaching susceptibility.

Assessing differential expression of the coral host under normoxic and deoxygenation conditions at the baseline temperature enabled identification of deoxygenation-induced stress processes that could otherwise be disguised under heating. Under deoxygenation (2 mg L^−1^ O_2_) and prior to the visible onset of bleaching, several genes involved in photoreception, such as rhodopsin, retinal pigment (RPE65), and retinol dehydrogenase, were downregulated. Rhodopsin downregulation has been observed for corals exposed to only deoxygenation^25^ or heating^35^, but how this potentially feeds into an altered capacity to sense and respond to light stimuli is currently unconsidered. In other animals, photoreceptors including rhodopsin, are known to be sensitive to hypoxia, as photoreceptor metabolism involves high levels of O_2_ consumption^90^. Consequently, rhodopsin activity and regeneration can be hindered by oxidative stress under hypoxia^91^, and such stress was evident in our deoxygenation treatment at the baseline temperature via significant upregulation of numerous antioxidants (e.g., sulfite oxidase and glutathione peroxidase) and pro-oxidants, such as D-amino acid oxidase, which generates hydrogen peroxide^92^. Consequently, it is plausible to hypothesise that an inability to trigger photoprotective mechanisms under high irradiance levels and limited O_2_ supply may be key in driving metabolic bleaching cascades. Specifically, irrespective of whether triggered by reduced external O_2_ availability or by an enhanced intra-tissue hypoxic state from high O_2_ demands under heat stress, a lack of photoprotection would presumably influence ‘optical feedback loops’ (e.g., loss of light capture by Symbiodiniaceae) that affect coral bleaching^93–95^. Evidence of oxidative stress under deoxygenation as observed here, even before the onset of visual bleaching, is consistent with the oxidative stress theory of coral bleaching^17,43,96^, although oxidative stress itself might be the consequence of further upstream dysbiotic events ^22,97^. Regardless of ROS being the cause or a consequence of bleaching, it highlights how hypoxia stress may be an overlooked but fundamental stressor to heat-induced coral bleaching (summarised in Fig. 4).

## Summary

We employed the Coral Bleaching Automated Stress System (CBASS) to show that deoxygenation can lower thermal stress thresholds of corals. Subsequent RNA-Seq analysis suggests a high degree of consistency in the coral stress response to heating with and without deoxygenation and key hypoxia-induced genes were differentially expressed under heating alone. This highlights the inherent link between heat stress and hypoxia at the molecular level. At the same time, we observed a specific gene expression response associated with deoxygenation that corroborates how heat stress is further exacerbated under low O_2_ levels, such as compromising photoprotective mechanisms. Thus, our results suggest that coral with a high tolerance to low O_2_ are likely more tolerant to heat stress. This study supports hypoxia as an overlooked factor in coral bleaching and further flags the need to establish routine O_2_ monitoring on coral reefs.

## Supplementary Information

Supplementary Information.

## Data Availability

Sequence data determined in this study are available under NCBI BioProject PRJNA808230 at https://www.ncbi.nlm.nih.gov/bioproject/?term=PRJNA808230. Scripts and input data used for RNA‐Seq data analysis and for DRC models are available at GitHub https://github.com/reefgenomics/CBASS_hypoxia.
