# Author Correction: Deoxygenation lowers the thermal threshold of coral bleaching

**DOI:** 10.1038/s41598-023-40318-y

**Published:** 2023-08-15

**Authors:** Rachel Alderdice, Gabriela Perna, Anny Cárdenas, Benjamin C. C. Hume, Martin Wolf, Michael Kühl, Mathieu Pernice, David J. Suggett, Christian R. Voolstra

**Affiliations:** 1 Climate Change Cluster, Faculty of Science, University of Technology Sydney, Ultimo, NSW 2007 Australia; 2 Department of Biology, University of Konstanz, 78457 Konstanz, Germany; 3 Marine Biology Section, Department of Biology, University of Copenhagen, Strandpromenaden 5, 3000 Helsingør, Denmark

Correction to: *Scientific Reports* https://doi.org/10.1038/s41598-022-22604-3, published online 31 Oct 2022

The original version of this Article contained errors in the Materials and methods section, where the Zenodo data repository link was incorrectly given as https://zenodo.org/deposit/6497221. Consequently, under the subheading ‘Coral bleaching assessment’,

“Photographs were taken after the 6 h stress phase (T1) and also prior to the CBASS experiment at T0 using the same camera settings, working distance, and illumination level (all images are available at https://zenodo.org/deposit/6497221;^58^).”

now reads:

“Photographs were taken after the 6 h stress phase (T1) and also prior to the CBASS experiment at T0 using the same camera settings, working distance, and illumination level (all images are available at https://zenodo.org/record/6497221;^58^).”

Under the subheading ‘De novo transcriptome assembly’,

“We consider the transcriptome of 20,115 contigs the reference transcriptome on which all expression analyses were based on, accessible at https://zenodo.org/deposit/6497221^58^;”

now reads:

“We consider the transcriptome of 20,115 contigs the reference transcriptome on which all expression analyses were based on, accessible at https://zenodo.org/record/6497221^58^;”

Under the subheading ‘ITS2-based Symbiodinaceae profiling’,

“Output data from SymPortal was then plotted in R. SymPortal output files are available at https://zenodo.org/deposit/6497221^58^.”

now reads:

“Output data from SymPortal was then plotted in R. SymPortal output files are available at https://zenodo.org/record/6497221^58^.”

In addition, Reference 58 was incorrectly given as:

58. Alderdice, R. *et al.* Data from ‘Deoxygenation lowers thermal threshold of coral bleaching.’ *Zenodo*. https://zenodo.org/deposit/6497221 (2022).

The correct reference is listed below:

58. Alderdice, R. *et al*. Data from ‘Deoxygenation lowers thermal threshold of coral bleaching.’ [Data set]. *Zenodo*. https://doi.org/10.5281/zenodo.6497221 (2022).

The original Article has been corrected.

