# Supplementary material for: Deoxygenation lowers the thermal threshold of coral bleaching
Source: Sci Rep. 2022 Oct 31;12:18273. doi: 10.1038/s41598-022-22604-3 (PMC9622859; doi:10.1038/s41598-022-22604-3)
Supplement: Supplementary file 1 — Supplementary Information. [file 41598_2022_22604_MOESM1_ESM.pdf]

**Supplemental Information**

**Title: Deoxygenation lowers the thermal threshold of coral bleaching.**


**Authors:**

Rachel Alderdice[1&2*], Gabriela Perna[2], Anny Cárdenas[2], Benjamin C.C. Hume[2], Martin Wolf[2], Michael Kühl[3], Mathieu Pernice[1], David J. Suggett[1], Christian R. Voolstra[2*]

**Affiliations:**

[1] University of Technology Sydney, Climate Change Cluster, Faculty of Science, Ultimo, New South Wales, 2007, Australia.

[2] Department of Biology, University of Konstanz, 78457 Konstanz, Germany.

[3] Marine Biology Section, Department of Biology, University of Copenhagen, Strandpromenaden 5, DK-3000 Helsingør, Denmark.

*Corresponding authors:

rachel.alderdice@uni-konstanz.de

christian.voolstra@uni-konstanz.de

**Supplementary Figures**

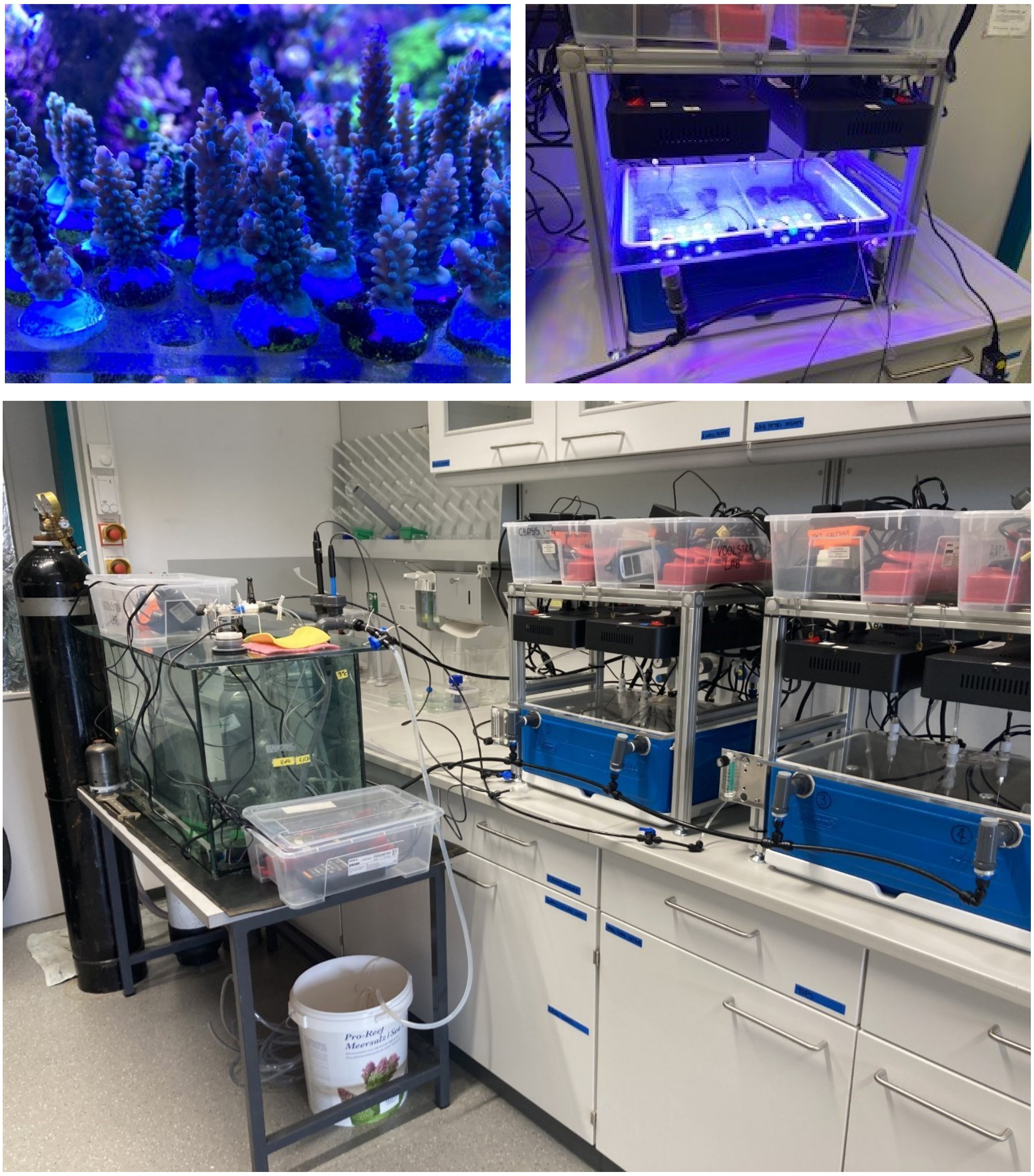


**Figure S1. Images of the experimental setup.** Shown are coral fragments positioned upright onto ceramic plugs (top left), the transparent lid used for sealing the CBASS tanks from exchange with ambient air (top right), and the overall setup including 4 CBASS tanks within 2 boxes and the reservoir tank.

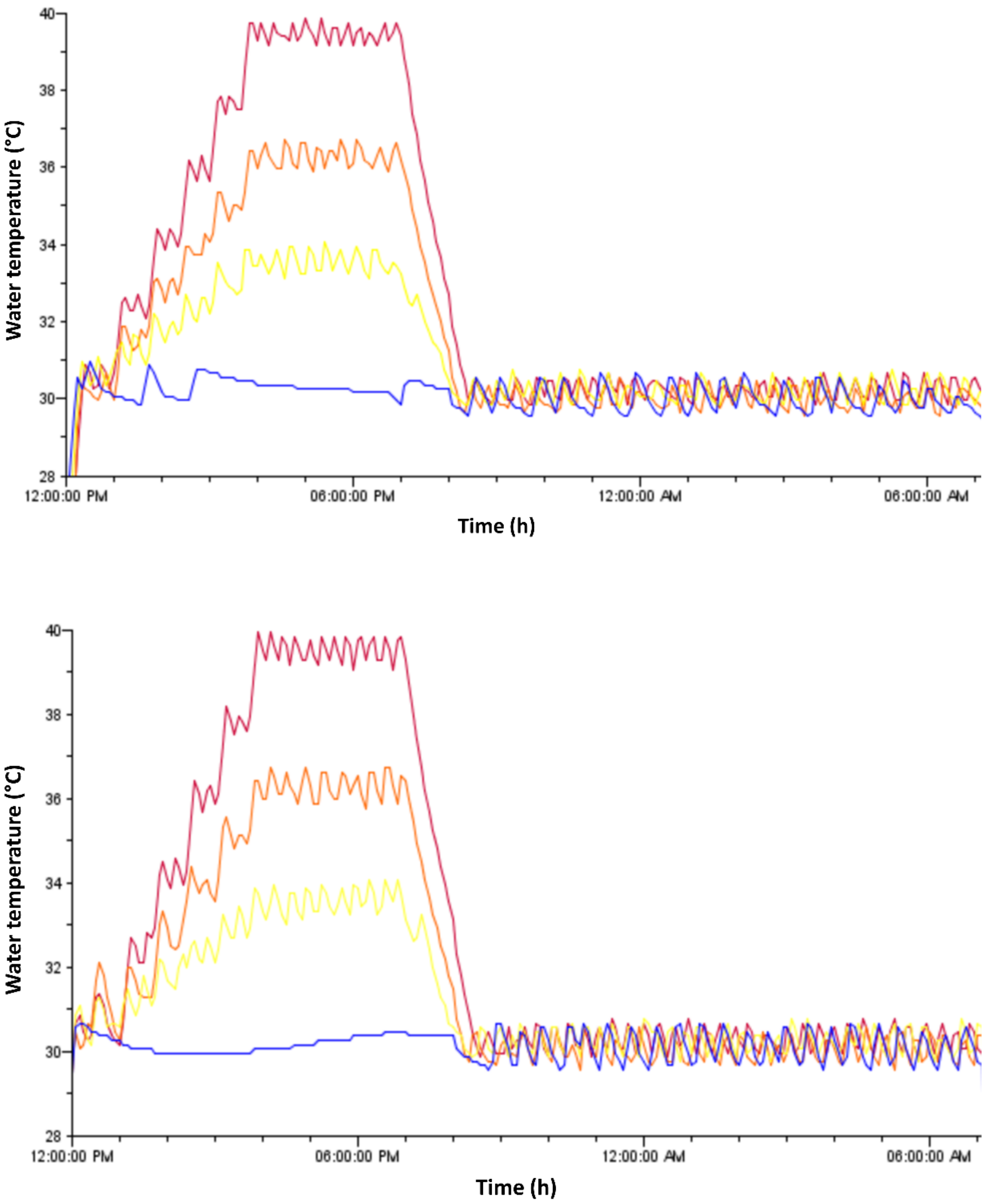


**Figure S2. Thermal profiles of CBASS runs.** HOBO logger data for each experimental tank of CBASS from 12pm until 7am the next day for the (i) heat only run on 31/05/2021 and (ii) the heat & deoxygenation run on 04/06/2021. Blue, yellow, orange, and red lines represent temperature data from tanks reaching 30°C, 33°C, 36°C, and 39°C, respectively.

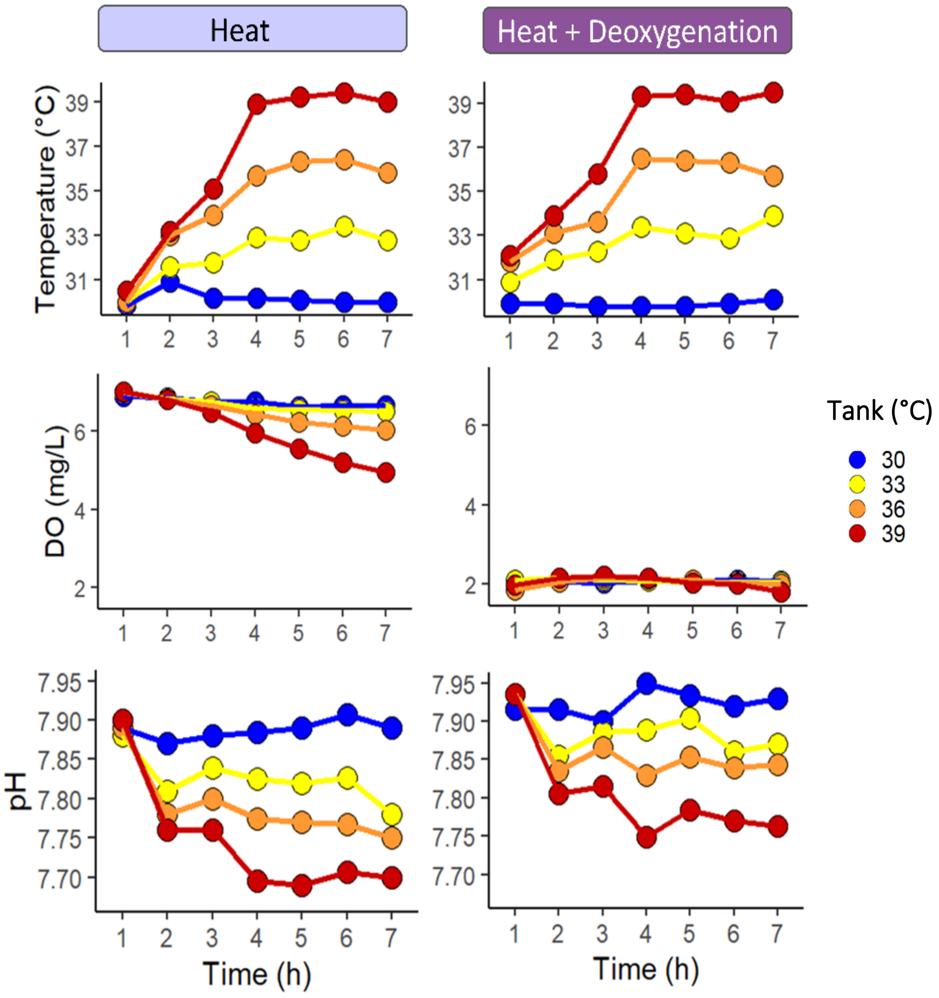


**Figure S3. Hourly measurements of temperature (°C), dissolved oxygen (DO), and pH during 6 h heat stress phase.** FireSting fibre-optic measurements using robust probes for each parameter during both CBASS runs, heat only on 31/05/2021 and heat & deoxygenation on 04/06/2021. Blue, yellow, orange, and red dot points represent temperature data from tanks reaching 30°C, 33°C, 36°C, and 39°C, respectively.

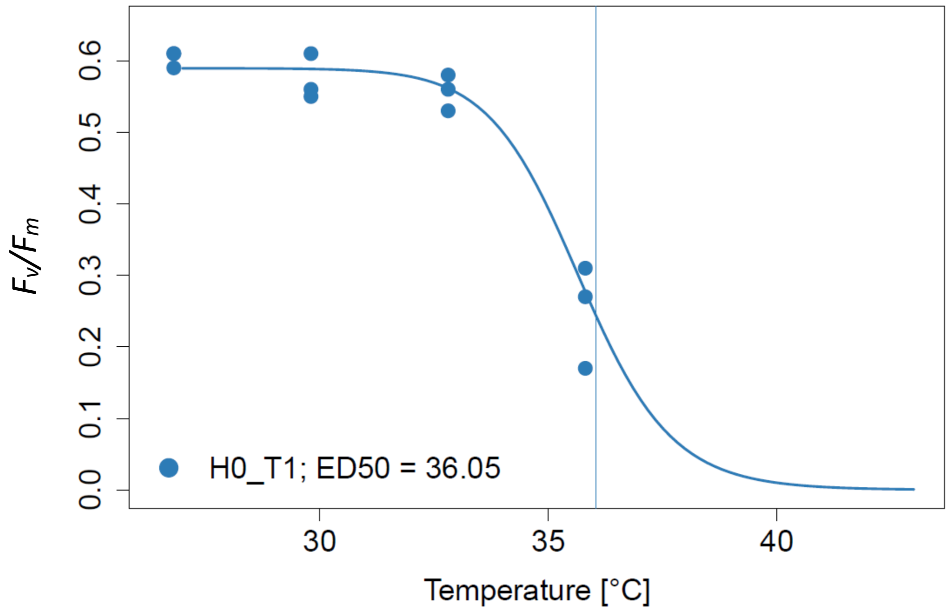


**Figure S4**. **ED50 based on photosynthetic efficiency (*$F_v$/$F_m$*) over lower temperature range with a baseline of 27°C.** The temperature at which the $F_v$/$F_m$ is reduced by 50% is the effective dose 50, ED50, of corals, a standardized thermal tolerance diagnostic (sensu Evensen et al., 2021). Sample replicates n= 3 for this trial run to assess the optimal temperature range to model the ED50. The curve reflects the mean three parameter log-logistic model. H0= Heat only and T1= time point 1. The projected ED50 is higher than the highest treatment temperature, suboptimal for accurate modelling.

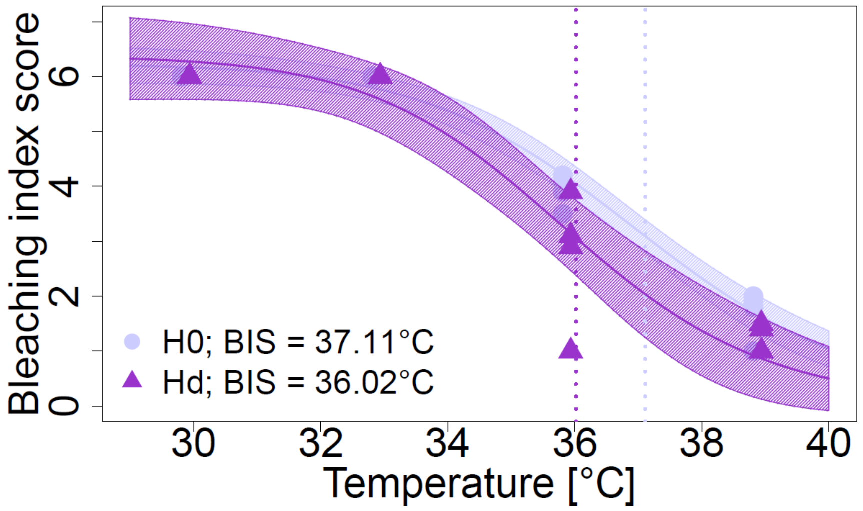


**Figure S5. ED50 thermal threshold based on Bleaching Index Score (BIS).** The temperature at which BIS is reduced by 50%, designates the effective dose 50, ED50 of corals, a standardized thermal tolerance diagnostic (sensu Evensen et al., 2021). Solid lines reflect the mean three parameter log-logistic model with 95% confidence intervals represented by the shaded areas. Hd= heat & deoxygenated conditions, H0= heat only indicated in dark and light purple respectively. n= 4 per heating tank.

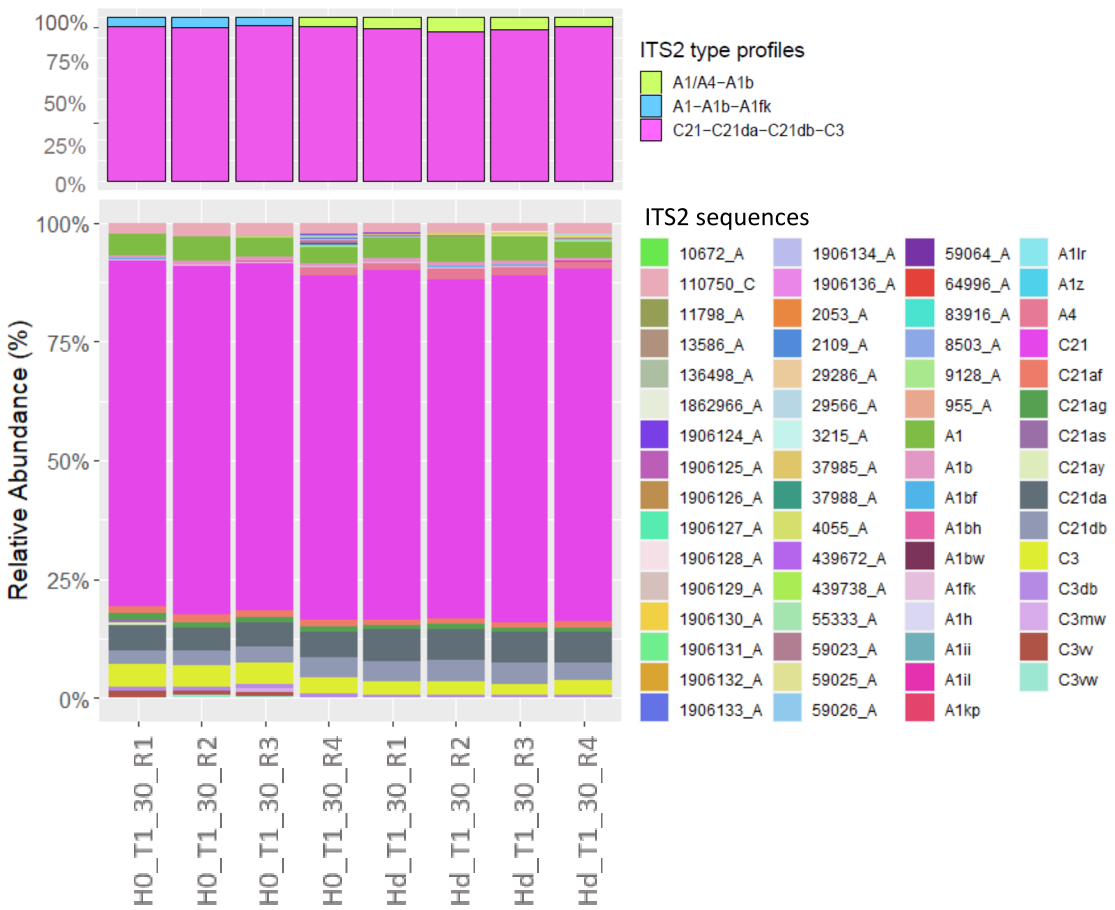


**Figure S6. Symbiodiniaceae assemblage of experimental corals**. Depicted are relative abundances of post-QC ITS2 sequences (lower panel) and predicted ITS2 type profiles (upper panel) processed by SymPortal for each coral sample at baseline temperature (30°C) from both heat only (H0) and heat & deoxygenation (Hd), n=4 per treatment.

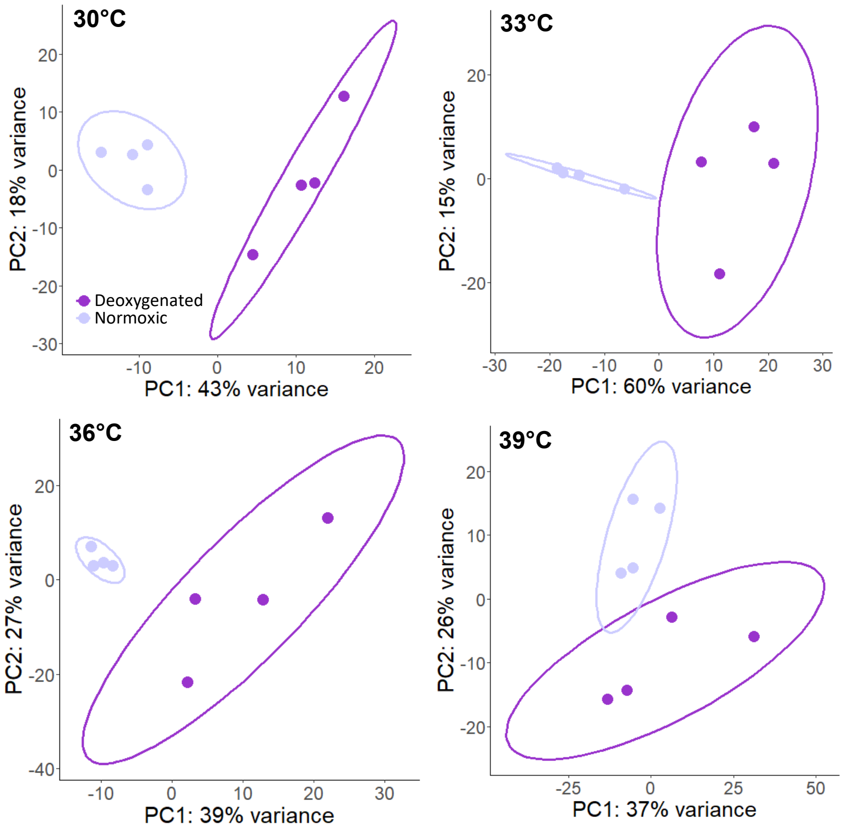


**Figure S7. Transcriptome clustering in response to normoxic/deoxygenation heat stress.** Similarity of *Acropora* sp. samples denoted by principal components analysis at four different temperatures (30°C, 33°C, 36°C, and 39°C). Dark and light purple represents deoxygenated and normoxic samples respectively. Circles denote 95% confidence level of dispersion estimates. Number of replicates per condition group n= 4.

## Supplementary Tables

**Table S1.** Aquarium holding tank parameters recorded on 18/05/2021 and based on 10+ years of consistent aquarium-rearing conditions.

| Parameters | Range |
|---|---|
| Dissolved oxygen | ~6-7mg/L |
| Salinity | 35 PSU |
| pH daily range | 7.7-8.2 |
| Temperature | 26.5°C (winter months =25°C) |
| Light intensity range | 95-240 µmol photons $m^{-2}$ $s^{-1}$ |
| Light spectrum | White & blue |

**Table S2. Read 1 count at different stages of the analysis pipeline.** These stages included trimming sequenced reads of Illumina adaptors and low-quality regions, mapping them to the de novo transcriptome assembly of the *Acropora* sp. (with a total of 20115 contigs) and filtering out samples < 5 million read counts. Sample ID represents the following: H0 = heat only (control), HH= heat & deoxygenation (a.k.a Hd, treatment), T1= time point 1, 30, 33, 36, 39 = temperatures of heat assay, and 1-4 = replicate number.

| **Sample ID** | **Initial** | **Trimming** | | **Mapping ≥ 500bp** | | **Quality Check** |
|---|---|---|---|---|---|---|
| | **Read count** | **Paired end read count** | **% Counts** | **Paired end reads mapped** | **% Mapping rate** | **≥ 5 million counts** |
| H0_T1_30_1 | 31429339 | 30464881 | 97 | 8510789 | 28 | y |
| H0_T1_30_2 | 32238151 | 31167654 | 97 | 8264998 | 27 | y |
| H0_T1_30_3 | 36506778 | 35219011 | 96 | 8819356 | 25 | y |
| H0_T1_30_4 | 30612235 | 29567727 | 97 | 7102331 | 24 | y |
| H0_T1_33_1 | 32145998 | 30958834 | 96 | 9052783 | 29 | y |
| H0_T1_33_2 | 29420253 | 28405849 | 97 | 8404714 | 30 | y |
| H0_T1_33_3 | 33421263 | 32271878 | 97 | 9794984 | 30 | y |
| H0_T1_33_4 | 32641996 | 31619512 | 97 | 9056917 | 29 | y |
| H0_T1_36_1 | 30269192 | 29290194 | 97 | 7466870 | 25 | y |
| H0_T1_36_2 | 32553871 | 31509750 | 97 | 8210718 | 26 | y |
| H0_T1_36_3 | 32448850 | 31461896 | 97 | 7560286 | 24 | y |
| H0_T1_36_4 | 31213926 | 30276231 | 97 | 7930491 | 26 | y |
| H0_T1_39_1 | 34280899 | 33089353 | 97 | 9083330 | 27 | y |
| H0_T1_39_2 | 31649570 | 30599193 | 97 | 8322156 | 27 | y |
| H0_T1_39_3 | 31163076 | 30183090 | 97 | 7907971 | 26 | y |
| H0_T1_39_4 | 34728077 | 33669153 | 97 | 8942517 | 27 | y |
| HH_T1_30_1 | 31344500 | 30280411 | 97 | 8549636 | 28 | y |
| HH_T1_30_2 | 31782681 | 30724004 | 97 | 8820920 | 29 | y |
| HH_T1_30_3 | 31607218 | 30533262 | 97 | 7558163 | 25 | y |
| HH_T1_30_4 | 32836621 | 31761101 | 97 | 8725054 | 27 | y |
| HH_T1_33_1 | 32141993 | 31022137 | 97 | 9797996 | 32 | y |
| HH_T1_33_2 | 33817095 | 32661920 | 97 | 11486096 | 35 | y |
| HH_T1_33_3 | 32908694 | 31822584 | 97 | 10350363 | 33 | y |
| HH_T1_33_4 | 33312555 | 32144454 | 96 | 10345889 | 32 | y |
| HH_T1_36_1 | 31863811 | 30752335 | 97 | 8388277 | 27 | y |
| HH_T1_36_2 | 31135202 | 30329344 | 97 | 8160821 | 27 | y |
| HH_T1_36_3 | 33050565 | 31954907 | 97 | 8687879 | 27 | y |
| HH_T1_36_4 | 30672997 | 29621039 | 97 | 9444586 | 32 | y |
| HH_T1_39_1 | 31566009 | 30418097 | 96 | 7099524 | 23 | y |
| HH_T1_39_2 | 31871308 | 30707770 | 96 | 7557541 | 25 | y |
| HH_T1_39_3 | 31108401 | 29931191 | 96 | 5378213 | 18 | y |
| HH_T1_39_4 | 30272993 | 29133359 | 96 | 7770008 | 27 | y |
| Av. | 32125504 | 31048504 | 97 | 8517256 | 27 | 32/32 |

**Table S3. Number of unique contigs per taxa group in de novo transcriptome assemblies.** Coral holobiont taxa contributions of total contigs in percentage (%) based on BLAST query results of transcriptome assemblies when using all samples or only non-bleached samples (those at temperature 30°C and 33°C for both CBASS runs of heat only and heat with deoxygenation). Coral holobiont taxa groups of cnidaria, Dinophyceae, bacteria, fungi, virus and then other taxa were also considered. Number of contigs were also subset based on contig length. For this study the de novo transcriptome assembly used included non-bleached samples ≥ 500 bp from all taxa groups.

| | **Taxa group** | **≥0bp** | **% Taxa** | **≥500bp** | **% Taxa** | **% Removed** |
|---|---|---|---|---|---|---|
| ALL samples | Cnidaria | 101367 | 36 | 10861 | 69 | 89 |
| | Dinophyceae | 1090 | 0.38 | 170 | 1.08 | 84 |
| | Bacteria | 46802 | 16 | 1120 | 7 | 98 |
| | Fungi | 7010 | 2 | 263 | 2 | 96 |
| | Virus | 160 | 0.06 | 4 | 0.03 | 98 |
| | sum | 156429 | 55 | 12418 | 79 | |
| | other taxa | 128774 | 45 | 3264 | 21 | |
| | Total contigs | 285203 | 100 | 15682 | 100 | 95 |

| | **Taxa group** | **≥0bp** | **% Taxa** | **≥500bp** | **% Taxa** | **% Removed** |
|---|---|---|---|---|---|---|
| non-bleached samples | Cnidaria | 86210 | 74 | 17975 | 89 | 79 |
| | Dinophyceae | 974 | 0.83 | 441 | 2.19 | 55 |
| | Bacteria | 6362 | 5 | 284 | 1 | 96 |
| | Fungi | 2639 | 2 | 230 | 1 | 91 |
| | Virus | 47 | 0.04 | 6 | 0.03 | 87 |
| | sum | 96232 | 82 | 18921 | 94 | |
| | other taxa | 20837 | 18 | 1194 | 6 | |
| | Total contigs | 117069 | 100 | 20115 | 100 | 83 |

**Table S4. De novo transcriptome assembly statistics when using all samples or only non-bleached samples.** Statistics considering scaffolds and contigs based on SOAPdenovo-Trans statistic report and QUAST results. Non-B = non-bleached samples. Scaff. = scaffolds.

| | Total scaff. ≥ 0bp | Total scaff. ≥500bp | % Scaff. ≥500bp | GC % | Median scaff. length | Av. scaff. length | Total bases | N50 ≥ 100bp | N50 ≥ 500bp |
|---|---|---|---|---|---|---|---|---|---|
| **ALL** | 2096835 | 230724 | 11 | 48 | 209 | 321 | 674 M | 368 | 1442 |
| **Non-B** | 844774 | 139488 | 17 | 45 | 183 | 413 | 349 M | 854 | 1946 |

| | Total contigs ≥0bp | Total contigs ≥500bp | % contigs ≥500bp | GC % | Median contig length | Av. contig length | Total bases | N50 ≥ 100bp | N50 ≥ 500bp |
|---|---|---|---|---|---|---|---|---|---|
| **ALL** | 2508117 | 182540 | 7 | 45 | 252 | 158 | 634 M | 390 | 1946 |
| **Non-B** | 1018615 | 121034 | 12 | 45 | 302 | 171 | 308 M | 390 | 1946 |

**Table S5. Number of differentially expressed transcripts between different temperatures.** Differential expression based on Benjamini-Hochberg FDR correction (p adj. <0. 05). Comparisons show Hd = deoxygenated treatment and H0 = control samples for each temperature (33, 36, 39°C) versus control of 30°C (baseline temperature). n = number of samples in total for treatment (n=4) and control (n=4). Number of annotated differentially expressed transcripts based on 6556 annotations from EggNOG Orthology.

| **Comparison** | **Total n** | **Total Upreg.** | **Total Downreg.** | **Annotated Upreg.** | **Annotated Downreg.** |
|---|---|---|---|---|---|
| 33H0 vs 30H0 | 8 | 4408 | 4396 | 1655 | 2199 |
| 33Hd vs 30Hd | 8 | 3841 | 3831 | 1333 | 1910 |
| 36H0 vs 30H0 | 8 | 5638 | 6715 | 2047 | 2980 |
| 36Hd vs 30Hd | 8 | 5341 | 6322 | 2777 | 1956 |
| 39H0 vs 30H0 | 8 | 4995 | 5807 | 1768 | 2676 |
| 39Hd vs 30Hd | 8 | 4562 | 5726 | 1551 | 2632 |

**Table S6. Number of differentially expressed transcripts in pairwise comparisons between normoxia and deoxygenation for each temperature.** Number of differentially expressed transcripts following Benjamini-Hochberg FDR correction (p adj. <0. 05). Comparisons show Hd = deoxygenated treatment and H0 = control samples for each temperature (temp.; °C). n = number of samples in total for treatment (n=4) and control (n=4). Number of annotated differentially expressed transcripts based on 6556 annotations from EggNOG Orthology.

| **Comparison** | **Temp. (°C)** | **Total n** | **Total Upreg.** | **Total Downreg.** | **Annotated Upreg.** | **Annotated Downreg.** |
|---|---|---|---|---|---|---|
| Hd vs H0 | 30 | 8 | 382 | 491 | 152 | 209 |
| Hd vs H0 | 33 | 8 | 1134 | 1137 | 399 | 594 |
| Hd vs H0 | 36 | 8 | 187 | 356 | 81 | 154 |
| Hd vs H0 | 39 | 8 | 130 | 73 | 52 | 31 |
